# Impact of Cytochrome P450 Enzyme on Fruit Quality

**DOI:** 10.3390/ijms25137181

**Published:** 2024-06-29

**Authors:** Daniela Minerdi, Paolo Sabbatini

**Affiliations:** 1 Department of Agricultural, Forestry and Food Sciences, University of Turin, Largo Paolo Braccini 2, 10095 Grugliasco, TO, Italy; paolo.sabbatini@unito.it; 2 Department of Horticulture, Michigan State University, East Lansing, MI 48824, USA; 3 Interdepartmental Centre for Grapevines and Wine Sciences, University of Turin, Corso Enotria 2/C, 12051 Alba, CN, Italy

**Keywords:** cytochromes P450 enzymes, plants, fruit quality, fruit ripening, grape berries

## Abstract

Cytochrome P450 enzymes are monooxygenases widely diffused in nature ranging from viruses to man. They can catalyze a very wide range of reactions, including the ketonization of C–H bonds, N/O/S-dealkylation, C–C bond cleavage, N/S-oxidation, hydroxylation, and the epoxidation of C=C bonds. Their versatility makes them valuable across various fields such as medicine, chemistry, and food processing. In this review, we aim to highlight the significant contribution of P450 enzymes to fruit quality, with a specific focus on the ripening process, particularly in grapevines. Grapevines are of particular interest due to their economic importance in the fruit industry and their significance in winemaking. Understanding the role of P450 enzymes in grapevine fruit ripening can provide insights into enhancing grape quality, flavor, and aroma, which are critical factors in determining the market value of grapes and derived products like wine. Moreover, the potential of P450 enzymes extends beyond fruit ripening. They represent promising candidates for engineering crop species that are resilient to both biotic and abiotic stresses. Their involvement in metabolic engineering offers opportunities for enhancing fruit quality attributes, such as taste, nutritional content, and shelf life. Harnessing the capabilities of P450 enzymes in crop improvement holds immense promise for sustainable agriculture and food security.

## 1. General Features of Cytochrome P450 Enzyme

Cytochrome P450 enzymes (CYPs) belong to an ancient supergene family that is extensively spread across nature, spanning from viruses to humans; they showcase remarkable diversity, both in terms of sequence and biochemical characteristics [1]. P450 enzymes possess a heme prosthetic group, and they need electron transfer partners to deliver electrons to the iron located in the heme from the cofactor during catalysis. P450s are divided into ten classes, from class I to class X [2], according to the kind of electron transfer partners and their structural organization. P450 enzymes show a high absorbance peak at 450 nm when they combine with reduced CO. They have a conserved aminoacid sequence in the heme-binding domain (Phe-X-X-Gly-X-Arg-X-Cys-X-Gly), K-helix (Glu-X-X-Arg), I-helix (Ala/Gly-Gly-X-Asp/Glu-Thr/Ser), and PxRx (Pro-X-Arg-X) [3]. P450 proteins all have a common catalytic centre, which is a conserved cysteine residue capable of binding to the heme iron [4]. The predominant CYPs typically function as monooxygenases, initiating the binding and reductive activation of molecular oxygen. This process results in the splitting of oxygen into its constituent atoms, with one atom being incorporated into the organic substrate RH held within the P450 active site. The majority of P450 reactions involve oxidations, utilizing one oxygen atom to generate the product ROH while concurrently reducing the second oxygen atom to water. This process adheres to the general reaction pattern as follows:

NAD(P)H + O_2_ + RH + H^+^ → NAD(P)^+^ + ROH + H_2_O

P450 enzymes use the pyridine nucleotide Nicotinamide Adenine Dinucleotide (NADH) or Nicotinamide Adenine Dinucleotide (NADPH) as a cofactor to deliver electrons via a flavoprotein. The direct reaction of oxygen in the triplet state with singlet organic molecules is spin-forbidden [5], but P450 enzymes overcome this barrier by complexing the oxygen to iron, so that the metal–oxygen complex can react with carbon substrates. P450 enzymes play a crucial role in the biosynthesis of a diverse range of primary and secondary metabolites through various enzymatic processes, including C-H bond hydroxylation, N-dealkylation, N-hydroxylation, isomerization, O-dealkylation, S-oxidation, reduction, desaturation, decarboxylation, dimerization, epoxidation, C–C cleavage, and ring extension reactions [6].

### 1.1. Catalytic Cycle

**Figure 1:**
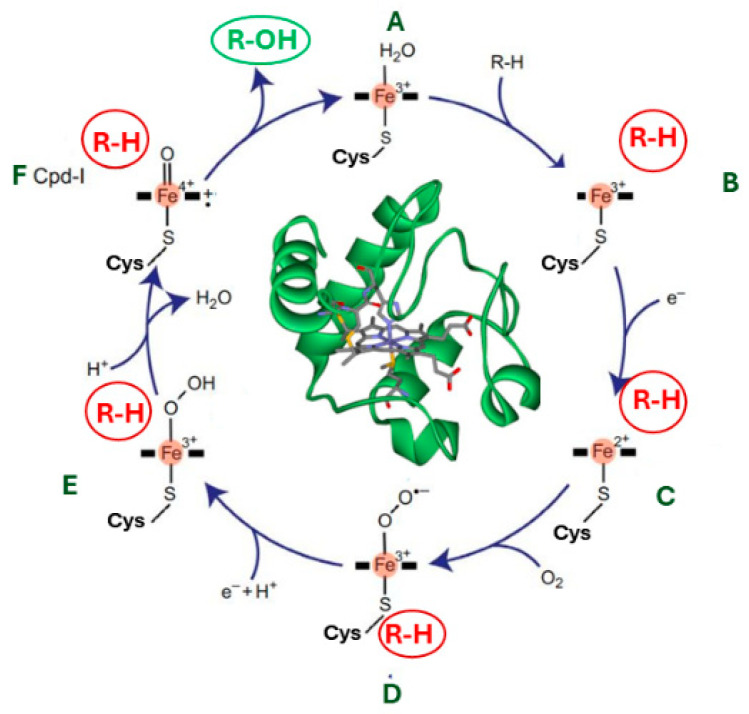
P450 enzymes catalytic cycle. Adapted from ref. [8]. Substrate binds the active site (**A**); electron transfer from NADH to FMN begins (**B**); iron changes from its ferric state (Fe^3+^) to its ferrous state (Fe^2+^) (**C**); molecular oxygen binds at the heme iron and activate the oxygen (**D**); in the second reduction phase an intermediate peroxo group is produced (**E**); Cytochrome P450 compound I (Cpd-I) is produced (**F**).

When the substrate (S) binds the active site in the heme domain (Figure 1A), one molecule of water is released, triggering a conformational shift in both the active site and the reductase domain. This shift brings the flavins into closer proximity, aiding in the electron transport process (Figure 1B) [7].Concurrently, the iron undergoes a transition from a low spin state to a high spin state. This alteration in energy levels results in the generation of a distinctive “difference spectra” when measuring the enzyme-substrate complex using a spectrophotometer. The absorbance at 390 nm increases while there is a decrease at 420 nm determining a difference spectrum. If carbon monoxide binds to a reduced P450, an absorbance shift to the characteristic 450 nm occurs, and the change during substrate binding in the presence of NAD(P)H, triggers the initial donation of primary electrons. The electron first transfers from NAD(P)H to the initial flavin molecule, then proceeds to FMN, and ultimately reaches the heme group, where it is received by the iron within the porphyrin ring. Iron changes from its ferric state (Fe^3+^) to its ferrous state (Fe^2+^) (Figure 1C). Once the initial reduction has occurred, molecular oxygen binds covalently at the distal axial position of the heme iron and activate the oxygen [7] (Figure 1D). During this phase, the dissociation of the iron–oxygen bond can generate a highly reactive superoxide radical, which has the potential to interfere with the catalytic cycle. Subsequently, in the second reduction step (Figure 1E), an intermediate peroxo group with a negative charge and high nucleophilicity is produced. It is protonated twice due to amino acid side chains and hydrogen binding from surrounding water. The dioxygen that binds to the donated hydrogen undergoes a reaction that results in the release of a water molecule, leading to the formation of the cytochrome P450 compound I intermediate (Figure 1E). This intermediate is characterized by an Fe^4+^ group, which arises from the electron pair donated by the nucleophilic peroxo group, and it contains a double-bonded oxygen atom. Cytochrome P450 compound I (Cpd-I) (Figure 1F) represents the final intermediate phase in the P450 catalytic cycle and is considered the main oxidizing agent responsible for producing the hydroxylated product [8].

### 1.2. P450 Classification

Redox protein partners supply the electrons necessary to sustain the P450 catalytic cycle, enabling the sequential delivery of two electrons to various intermediates within the cycle [9]. In the case of some P450s, their reductase comprises one or more distinct proteins. Alternatively, in some instances, the reductase domain is integrated with the P450 domain within a single polypeptide chain. At this point, the enzyme is self-sufficient and only NADPH addition is needed to trigger catalysis without adding separate proteins. Rarely P450s have been reported to achieve the reductive activation of oxygen without involving any redox partner [10]. P450 enzymes are classified into 10 distinct classes (from class I to class X), based on their redox partners and structural arrangement. From class I to VI, they need reductase partners. Class VII P450 enzymes comprises only soluble and cytosolic bacterial enzymes with a single polypeptide chain that includes a heme-containing P450 domain at the N-terminus and a Flavin Mono Nucleotide (FMN)-containing reductase domain at the C-terminus. The reductase domain is closely related to the phthalate oxidase reductase (PFOR) [11]. Class VII P450 can perform reactions in the presence of only the electron donor, without the need of a redox partner; for this reason they are called self-sufficient [2]. These enzymes can use thiocarbamate pesticides, aromatic compounds, and terpenes as substrates [12,13]. Class VIII includes cytosolic self-sufficient P450 enzymes, both prokaryotic and eukaryotic where a unique polypeptide chain harbors the heme domain at its N-terminus, while at the C-terminus there is a reductase domain akin to the eukaryotic cytochrome P450 reductase encompassing both Flavin Adenine Dinucleotide (FAD) and FMN subdomains. Class IX comprises nitric oxide reductase (P450nor) from the fungus *Fusarium oxysporum* CYP55 that contributes to the denitrification processes. This does not have monooxygenase activity, but possesses a unique electron transfer chain system. P450nor directly utilizes NADH as an electron donor, bypassing the requirement for a redox partner, to reduce two molecules of nitric oxide (NO) to nitrous oxide (N2O), thereby protecting the fungus from NO inhibition [14].

This reductive process in P450 reactivity is distinctive. Class X is also a P450-only system, but it utilizes acyl hydroperoxide, serving both as the oxygen donor and the substrate resulting in the formation of new carbon–oxygen bonds [15].

## 2. Roles of P450 Enzymes in Plants

Cytochromes P450 (CYPs) represent one of the largest protein families in plants, anchoring to the cytoplasmic surface of the endoplasmic reticulum via a hydrophobic peptide at the N-terminus. Their versatile functions encompass hormonal signaling [16], the biosynthesis of structural polymers [17], and inter-organism communication [18]. Crucially, P450 enzymes are indispensable for detoxifying xenobiotics and are pivotal in producing phytohormones, secondary metabolites, and antioxidants in higher plants. Additionally, these enzymes play a vital role in enhancing plant resilience to both biotic and abiotic stresses by scavenging reactive oxygen species (ROS), modulating phytohormone biosynthesis and homeostasis [19]. Specifically, P450 enzymes are involved in synthesizing antioxidants like tocopherols, carotenoids, and flavonoids, which are crucial for ROS scavenging during environmental stresses. Flavonoids, as polyphenols, intercept electrons from ROS, forming stable phenoxyl radicals that disrupt ROS-induced chain reactions within cell organelles [20,21]. Moreover, P450 enzymes contribute to plant defense against biotic stressors, including viruses, bacteria, fungi, nematodes, and insects, by synthesizing compounds such as cyanogenic glucosides, benzylisoquinoline alkaloids, isoflavonoids, glucosinolates, and diterpenoids [22]. Furthermore, plants employ an indirect defense mechanism against herbivores, releasing volatiles that repel herbivores and attract natural enemies of the feeding guilds. P450 enzymes participate in this process by synthesizing aldoximes and phenylacetaldoxime, components of the induced volatile blend that attract natural enemies, thereby reinforcing the indirect defense mechanism [23].

## 3. What Is Fruit Quality

The nutritional and health implications of fruit quality underscore its significance in human diets, heavily influenced by its biochemical composition dominated by low-molecular-weight metabolites. Fruit quality is the culmination of coordinated physiological processes spanning from anthesis through growth to ripening stages, subject to modulation by external factors including abiotic stressors and postharvest compositional changes [24]. Advanced biochemical analyses of the metabolome have been pivotal in unraveling fruit biochemical composition and metabolism, both pre- and post-harvest. These analyses often employ nuclear magnetic resonance (NMR) or mass spectrometry (MS)-based fingerprinting, profiling, or imaging techniques, frequently coupled with targeted analyses focusing on specific metabolites such as antioxidants [21]. Numerous case studies have explored metabolic reprogramming during fruit set, development, and postharvest stages, delving into environmental influences, gene function analysis, genetics, and systems biology [25]. Fruit quality encompasses sensory attributes and nutritional value intricately linked to their biochemical composition, particularly low-molecular-weight organic compounds (metabolites) like sugars, organic acids, amino acids, phenolic compounds, isoprenoids, and alkaloids, alongside minerals, starch, and cell walls. Ripe fruit composition reflects complex metabolic changes during development, with further modifications during postharvest stages including ripening, storage, and transportation [26]. Traditionally, targeted analyses focusing on specific biochemical families have dominated detailed metabolite measurements during fruit development. However, recent years have witnessed the rise of untargeted metabolomic approaches utilizing techniques such as proton NMR MS, often complemented by targeted methods. Model fruit metabolomics, notably in tomato, has paved the way for studying a diverse array of agriculturally significant fruit species. Fleshy fruits, intricate organs exhibiting variability in color, size, and shape, are composed of diverse tissues, the biochemical composition of which may vary significantly despite coordinated development. Thus, studying tissue-specific biochemistry through dissection or imaging techniques holds significance. Nearly a decade ago, Hanhineva and Aharoni [27] explored metabolomics’ application in tracking metabolism in transgenic plants, mutants, or populations, illuminating genomic regions associated with metabolite traits. While initially focused on tomato and strawberry, they predicted metabolomics’ expansion to encompass various other species, a foresight that has since materialized. Subsequent reviews on fruit metabolomics have underscored its contributions to understanding primary metabolism’s impact on fruit growth, development, and the evolution of specialized metabolic pathways across different developmental stages and environmental conditions [28].

### The Definition of Fruit Quality in Viticulture

The composition of wine encompasses over one thousand compounds, predominantly derived from grapes, including essential elements like vitamins and minerals, alongside products of the winemaking process such as ethanol and glycerol. The transformation of sugars significantly impacts wine quality, influencing both its alcoholic content and the activation of genes governing aromatic and sensory attributes [29]. Achieving physiological ripeness involves balancing sugar levels, acidity, and the presence of aromatic and phenolic compounds, as well as factors like berry softness and water content [30]. Assessing grape maturity, vital for wine quality, is complex due to diverse factors like grapevine variety, environmental conditions (e.g., soil, temperature, sun exposure), and hormonal influences. Ongoing research into grape ripening control mechanisms is imperative for enhancing grape and wine quality [31].

Grapes, belonging to *Vitis* spp., hold immense economic significance globally. In 2022, the vineyard area spanned over 7.28 million hectares, yielding over 74 million tons of grapes, ranking second in fruit production. Most of this production (about 70%) is allocated for wine, with 27% consumed fresh and 2% dried. Although over 50 species are recognized in the grape genus *Vitis*, *Vitis vinifera*, native to the Caucasus region, dominates global wine production [32]. Grape quality depends significantly on vineyard practices, with compounds produced by the plant influencing flavor and aroma. The transformation of sugars significantly impacts wine quality, influencing both its alcoholic content and the activation of genes governing aromatic and sensory attributes. Grape quality is crucial for the wine industry and is determined by various factors, including vineyard practices, terroir, and environmental conditions. “Terroir”, which encompasses soil, climate, and microorganisms, plays a pivotal role in grape quality. Climate, particularly temperature and rainfall, significantly impacts viticulture globally, with wine growers needing to adapt to natural conditions. The development and maturation of grape berries are subjects of scientific scrutiny due to their importance in both the human diet and the wine industry. *Vitis vinifera* is noted for its ability to store large quantities of sugar in its berries. Control of ripening timing, berry characteristics, acidity, and aroma compounds are vital concerns for viticulturists. Molecular and biochemical studies have been instrumental in understanding grape berry maturation and its impact on fruit and wine quality. By adjusting grape growing practices based on these insights, wine styles can be modified. Assessing grape maturity, which is vital for wine quality, is complex due to diverse factors like grapevine variety, environmental conditions (e.g., soil, temperature, sun exposure), and hormonal influences. Achieving physiological ripeness involves balancing sugar levels, acidity, and the presence of aromatic and phenolic compounds, as well as factors like berry softness and water content. Ongoing research into grape ripening control mechanisms is imperative for enhancing both grape and wine quality. The composition of wine encompasses over one thousand compounds, predominantly derived from grapes, including essential elements like vitamins and minerals, alongside products of the winemaking process such as ethanol and glycerol. Water constitutes the largest portion of wine (75–90%), with variations attributed to phenolics, organic acids, mineral salts, and pectins. Ethyl alcohol, the second significant constituent, ranges from 8% to over 13%, depending on the wine type. Sugar content differs widely, from under 2 g/L in dry wines to nearly 200 g/L in sweet varieties.

Grape quality depends significantly on vineyard practices, with compounds produced by the plant influencing flavor and aroma. “Terroir”, encompassing soil, climate, and microorganisms, plays a crucial role in grape quality. Climate, especially temperature and rainfall, profoundly impacts viticulture worldwide, with wine growers adapting to natural conditions. The development and maturation of grape berries are subjects of scientific scrutiny due to their importance in the human diet and the wine industry. *V. vinifera* is noted for its ability to store large quantities of sugar in its berries. Control of ripening timing, berry characteristics, acidity, and aroma compounds are vital concerns for viticulturists, with molecular and biochemical studies contributing to understanding grape berry maturation and its impact on fruit and wine quality. Adjusting grape growing practices based on these insights can modify wine style. The quality of wine relies not only on the presence of volatile compounds that affect aroma but also on phenolic compounds due to their influence on mouthfeel, color, flavor, and aging potential. These phenolic compounds, which are mainly found in the skins and seeds of grape berries, are extracted into the fermenting must (a mixture of grape solids and juice) during maceration. While every step in the winemaking process affects the final product, fermentation and the subsequent extraction of grape components are critical in adding value to the wine. The nutritional benefits of these grape components are significant. Phenolic compounds, such as flavonoids and tannins, contribute to the antioxidant properties of wine, which can offer health benefits when consumed in moderation. These antioxidants help to protect the body from oxidative stress and reduce the risk of chronic diseases. Additionally, the extraction process during fermentation enhances the concentration of these beneficial compounds in the final product Flavonoids encompass anthocyanidins, flavonols, flavanols, hydrolysable and condensed tannins, flavanones, flavones, and chalcones, each contributing uniquely to wine’s characteristics. Non-flavonoids include hydroxycinnamic acids, hydroxybenzoic acids, stilbenes, tyrosol, and hydroxytyrosol, further enriching wine profiles. Careful management of phenolic composition is pivotal for crafting wines with the desired attributes. Furthermore, precise extraction methods and analytical approaches play crucial roles in detecting and quantifying polyphenols, ensuring meticulous control over their concentrations in the final wine.

## 4. P450 Enzymes and Fruit Quality

In this section, we will explore the significant role of cytochrome P450 enzymes in shaping fruit quality across a diverse array of fruit species, including olive, apple, banana, durian, avocado, and tomato. We will pay particular attention to grape berries due to their agricultural and commercial importance. Cytochrome P450 enzymes play multifaceted roles in fruit development, ripening, and biochemical composition. Understanding these processes is essential for enhancing fruit breeding programs, optimizing cultivation practices, and developing innovative strategies to improve fruit quality, nutritional value, and consumer satisfaction.

### 4.1. Olive

**Figure 2:**
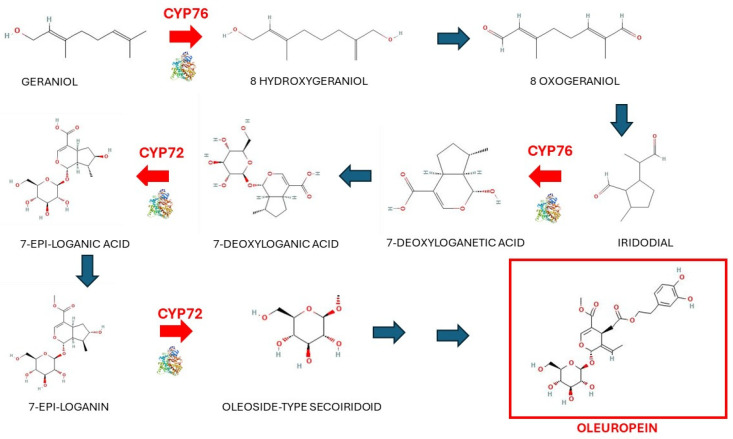
P450 enzymes contribute to the synthesis of Oleuropein in olive plants. Adapted from ref. [35].

Olive cultivation holds significant importance throughout Europe, boasting cultural heritage, economic value, and nutritional benefits. Extracted from the fruit, olive oil is a product abundant in essential nutrients and bioactive compounds. The flavor and quality of olive oil are tied to phenolic secoiridoids, specialized metabolites found within both the olive fruit and its oil, offering a spectrum of health advantages [33,34]. These secoiridoids stem from iridoids, a class of monoterpenes, by the cyclopentane ring opening process. Within the Oleaceae family, most iridoids transform into secoiridoids through the deoxyloganic acid pathway. Notably, oleuropein, a phenolic secoiridoid derivative, significantly influences olive products due to its inherent bitterness, a sought-after attribute in olive oil. Synthesized through the condensation of secoiridoid and hydroxytyrosol within the olive plant, the process of oleuropein’s formation is illustrated in Figure 2 [35].

### 4.2. Apple

**Figure 3:**
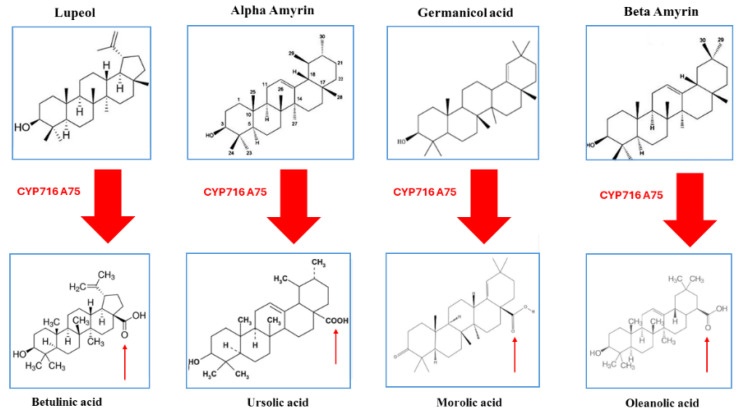
Contribution of P450 enzymes to the biosynthetic pathway of triterpenic acids in apple. Adapted from ref. [37].

The peels of fruits harbor a wealth of triterpenic acids known for their diverse beneficial effects, including antitumor, antioxidant, anti-inflammatory, antidiabetic, and hepatoprotective properties. Pentacyclic triterpenes, comprising six isoprene units, represent a significant class within this group. These triterpenes exhibit structural diversity, with the ursane, oleanane, and lupane series being the most prevalent among the pentacyclic triterpenes, deriving from α-amyrin, β-amyrin, and lupeol, respectively, marking their widespread distribution in nature [36]. Functionally, they contribute to plant resilience by safeguarding against water loss and various biotic and abiotic stresses, often in conjunction with other plant cuticle components. Within apples, bioactive ursane-, oleanane-, and lupane-type triterpenes are notably accumulated in the fruit cuticle. Research by Andre and colleagues [37] delved into their biosynthetic pathways, revealing the pivotal role of CYP716A175 in catalyzing the C-28 oxidation of α-amyrin, β-amyrin, lupeol, and germanicol. This enzymatic process yields ursolic acid, oleanolic acid, betulinic acid, and potentially morolic acid, as illustrated in Figure 3.

### 4.3. Banana

Bananas rank as the world’s second most significant fruit crop, providing a crucial source of carbohydrates for millions, particularly in developing regions. The expression patterns of the CYP71NI1 gene exhibit notable variations across different banana organs. Specifically, transcripts are exclusively detected in the peel and pulp of ripening fruit, absent in unripe fruit tissues across all developmental stages as well as in leaf, root, flower, and ovary tissues [38]. Throughout ripening, transcript levels remain low in pre-climacteric and climacteric fruits, gradually increasing and peaking in post-climacteric stages. Notably, the expression of CYP71N1 in pre-climacteric fruit can be stimulated by exogenous ethylene application and sucrose treatment of overripe fruit, suggesting a regulatory role of ethylene and/or sucrose at the transcript level in ripening associated P450 enzyme expression [38]. Despite these observations, the specific function of CYP71 in bananas remains unclear. De Bruyn and colleagues [39] elucidated the involvement of CYP71 in sesquiterpene lactone synthesis in chicory and witloof, hinting at its potential role in banana.

### 4.4. Tomato

**Figure 4:**
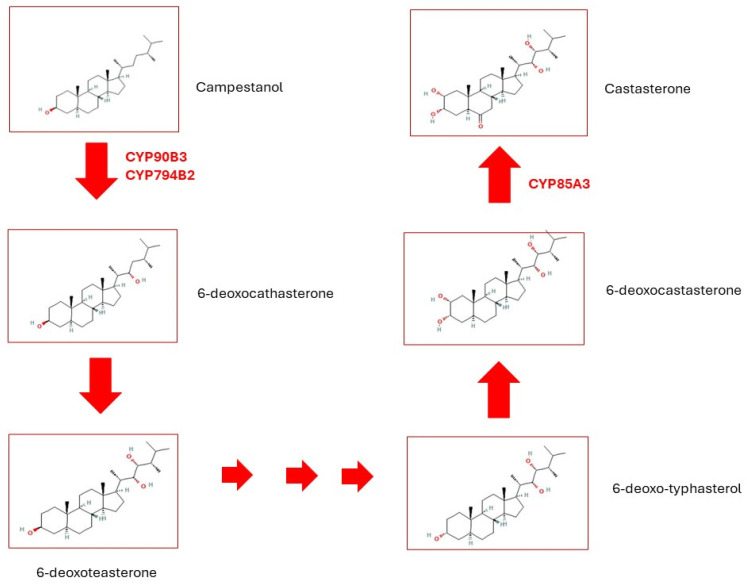
Synthesis of Castasterone. Campestanol in the C-6 oxidation pathway is oxidized to 6-deoxocathasterone which then is oxidized to generate 6-deoxoteasterone6-deoxotyphasterol, 6-deoxocastasterone, and castasterone. Adapted from ref. [43].

Tomatoes stand as a pivotal agricultural crop species, wherein fruit ripening hinges upon pigment accumulation, textural alterations due to tissue softening, and the enhancement of nutrients and flavors via the metabolism of bioactive compounds, sugars, acids, and volatile organic compounds. Carotenoids emerge as the primary pigments in mature tomato fruits, offering antioxidant defense against chronic ailments. Throughout ripening, sucrose, fructose, and glucose emerge as the predominant sugars [40]. Volatile compounds, derived predominantly from fatty acids, aliphatic amino acids, phenolic compounds, and carotenoids, comprise a diverse array, encompassing hydrocarbons, alcohols, aldehydes, esters, ethers, ketones, phenols, and compounds containing sulfur and nitrogen [40]. Notably, carotenoid-derived volatiles often exert positive influences on tomato flavor and consumer preference [41]. While the role of ethylene in fruit ripening is extensively documented, the involvement of brassinosteroids (BR) in ripening regulation and their interplay with the ethylene pathway remain less elucidated. Brassinosteroids, a class of polyhydroxylated steroidal phytohormones, are indispensable for plant development, growth, and productivity, orchestrating cell division, elongation, and differentiation across various cell types throughout the plant lifecycle [42]. Hu and colleagues uncovered active synthesis of BRs during tomato fruit ripening, with CYP90B3 catalyzing the pivotal rate-limiting step (Figure 4). The expression levels of this P450 enzyme positively correlate with tomato fruit ripening, concomitant with heightened softening and increased soluble sugar and flavor volatile contents. Over 70 natural BRs have been identified in plants, with castasterone emerging as widely distributed and the most bioactive among them. CYP90B3, CYP724B2, and CYP85A3 participate in their synthesis, commencing from the sterol campastenol [43] (Figure 4).

### 4.5. Durian

Durian, a fruit indigenous to Southeast Asian nations, carries substantial economic significance within the region. In a study conducted by Suntichaikamolkul and colleagues [44], P450 enzymes potentially linked to durian fruit ripening were identified. Their research revealed that the expressions of CYP88, CYP83, CYP707, CYP94, CYP72, and CYP714 were susceptible to external treatment with ripening regulators, indicating potential interactions among phytohormones in regulating fruit ripening. Notably, the expression levels of CYP707, CYP88, and CYP94, which are associated with jasmonic acid metabolism, differed significantly between fast- and slow-post-harvest ripening cultivars, implying crucial roles for these hormones in the ripening process. These phytohormone-related P450 enzymes may act as additional molecular regulators governing ripening mechanisms, complementing the functions of ethylene and auxin, thus contributing to the economically vital traits of durian fruit.

## 5. Cytochromes P450 in Grapevine

The genome of *V. vinifera* harbors 579 P450 sequences, among which 279 are complete genes exhibiting highly specific expression patterns. This diversity suggests a multifaceted role for P450 enzymes in various physiological processes of the grapevine. Some of these genes exhibit induction upon encountering biotic stress, indicating their involvement in defense mechanisms, while others are specifically activated during grape berry ripening. These latter genes may play pivotal roles in the synthesis of berry-specific metabolites, such as aroma compounds, which significantly contribute to the sensory qualities of grapes and wines [45,46]. In this section, we will delve into the intricate dynamics of P450 enzyme gene expression throughout the developmental stages leading up to grape berry ripening. By elucidating the temporal and spatial patterns of P450 enzyme gene expression, we aim to shed light on their potential roles in shaping the biochemical composition and sensory attributes of ripening grapes.

## 6. Grapevine

Grapevines (*Vitis* spp.) hold a significant position in both agricultural and cultural landscapes worldwide. Renowned for their versatile uses in winemaking, fresh fruit consumption, and as sources of phytochemicals, grapevines have long been cultivated for their economic importance. Beyond their role as a staple crop in viticulture, grapevines serve as models for studying fruit development, ripening processes, and the intricate biochemical pathways that underpin fruit quality traits [47]. Understanding the mechanisms governing grapevine fruit quality is not only essential for optimizing grape production but also for enhancing the sensory attributes, nutritional content, and market value of grapes and their derived products.

### Grape Berry Development and Ripening

Grape berries are composed of three primary tissues: skin, flesh, and seeds. As grapes progress through maturity, they undergo transformations in size, composition, color, texture, flavor, and susceptibility to pathogens. Typically, grape berries exhibit a double sigmoid growth pattern, initially propelled by cell division and later by cell expansion. The initial growth phase, extending from flowering to approximately 60 days thereafter, witnesses rapid berry formation alongside the development of seed embryos. This period sees the accumulation of various solutes, pivotal for berry expansion, peaking around 60 days post-flowering. Among these compounds, tartaric and malic acids predominate, playing a crucial role in determining acidity, a fundamental aspect of wine quality. Furthermore, hydroxycinnamic acids and tannins accrue, influencing browning reactions and the synthesis of aroma compounds. Minerals, amino acids, micronutrients, and aroma compounds also amass during this period, exerting profound effects on grape and subsequent wine quality. Subsequent to the initial growth phase, most grape cultivars enter a lag phase before embarking on a second growth phase, which heralds the onset of ripening, marked by véraison, the alteration in berry skin color. The most substantial alterations in grape composition occur during this ripening phase, wherein berries evolve from small, firm, acidic entities to larger, softer, sweeter, and more flavorful ones. Flavor progression primarily arises from the equilibrium between acidity and sugars, coupled with the synthesis of flavor compounds, significantly influencing the ultimate quality of wine produced. Throughout ripening, the berry’s size approximately doubles, with many solutes amassed during the initial growth phase retained but significantly diluted owing to the increased berry volume. Certain compounds, including malic acid, tannins, and specific aromatic compounds, diminish per berry during ripening. Notably, the ripening phase witnesses a substantial surge in glucose and fructose levels, indicative of a biochemical shift towards fruit ripening.

## 7. Expression of P450 Enzymes Genes in Berry

### 7.1. Early Period of Berry Development

During the initial phases of berry development, the expression of several key P450 enzymes genes undergoes notable changes. Specifically, genes such as CYP51, CYP71, CYP734A1, CYP90A1, CYP94C1a, and CYP710A1 demonstrate an upregulation in expression levels. These genes are likely involved in various metabolic processes crucial for early berry development, including hormone biosynthesis, cell wall modification, and defence responses [46]. However, as the fruit approaches maturity, the expression of these genes tends to decrease, indicating a shift in metabolic priorities towards processes associated with ripening and maturation. Understanding the temporal regulation of these P450 enzymes genes provide valuable insights into the molecular mechanisms underlying grape berry development and ripening.

#### 7.1.1. Fruit Development at Green Berry Stage

CYP82C2, CYP714A1, CYP704A1, CYP96A1, and CYP711A exhibit preferential expression at elevated levels in large, green-stage berries [46]. This heightened expression suggests potential roles for these P450 enzymesgenes in the biochemical processes underlying the growth and development of berries during this phase. Specifically, they may participate in pathways related to the biosynthesis of secondary metabolites, hormone signalling, or structural components crucial for berry enlargement and maturation.

#### 7.1.2. Ripening Process

The transcripts of CYP72A11, CYP716A1, and CYP87A2 genes exhibit upregulation during the ripening process of grapes [46]. This upregulation suggests that these specific P450 enzymesgenes play crucial roles in the biochemical transformations occurring as the berries progress towards maturation. It is likely that they are involved in the synthesis of ripening-related metabolites, including aroma compounds, pigments, and flavour precursors, which contribute to the sensory attributes and quality of ripe grapes and derived products like wine.

### 7.2. Role of P450 Enzyme in Wine Aroma

**Figure 5:**
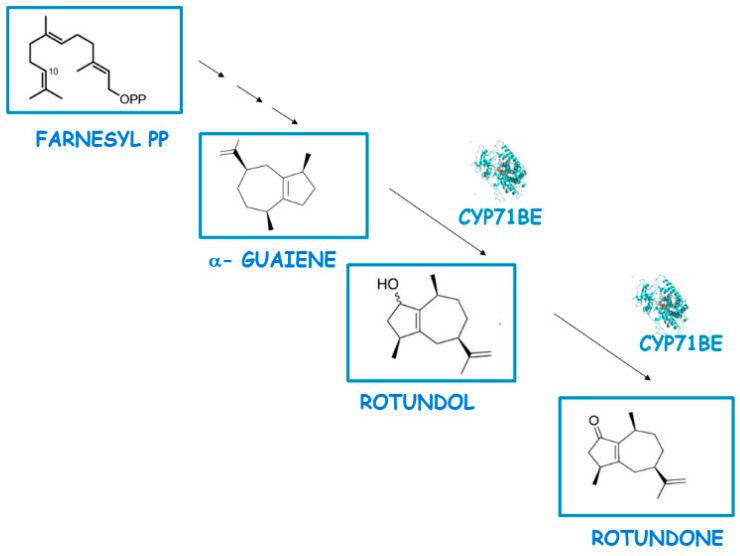
Contribution of CYPs to the synthesis of rotundone. Adapted from ref. [51].

Wine flavour is complex, involving hundreds of volatile molecules; among them, monoterpenoids, norisoprenoids, volatile sulphur compounds, and methoxypyrazines are the most important. These compounds are derived from secondary metabolites produced in grapevines and can be released during winemaking and aging through chemical and enzymatic reactions. Moreover, the relative concentrations of these compounds in grape berries are influenced by the grape variety as well as by various factors such as environmental conditions and the timing of the harvest [48]. Rotundone, an oxygenated sesquiterpene, is a key compound that significantly contributes to the spicy characteristic of wines and grapes. It was first identified in 2008 in red wine made from the Syrah grape cultivar [49]. Rotundone has since been found in several grape varieties and detected in the exocarp, stem, and leaf tissues [50]. Research by Takase et al. identified CYP71BE as a crucial enzyme in the rotundone biosynthesis pathway in grapevines, which oxidizes α-guaiene at C2, leading to the formation of rotundone [51]. The CYP71 clan encompasses more than 50% of all plant CYPs and exhibits a wide range of functions, including the oxidation of monoterpenes and sesquiterpenes in plant terpenoid metabolism [52] (Figure 5).

### 7.3. Role of P450 Enzymes in Shoot Elongation

Vine vigor, or vegetative growth, is a crucial factor influencing berry quality, which ultimately impacts the overall quality of wine produced. Recent research by Enoki and colleagues [53] has highlighted the importance of the enzyme CYP90D1 in this context. CYP90D1 is involved in the biosynthesis of brassinosteroids, which are plant hormones that regulate growth and development. The study demonstrated that CYP90D1 plays a significant role in shoot elongation, a key aspect of vegetative growth. The researchers conducted an RNA sequencing analysis on shoots collected seven days after bud break. This analysis revealed that CYP90D1 expression levels were highest in meristems, which are regions of active cell division, followed by internodes and leaves. This suggests that CYP90D1 is most active in the parts of the plant where growth is occurring most rapidly. Further experiments showed that vegetative growth and endogenous brassinolide content were significantly elevated in Arabidopsis plants overexpressing CYP90D1 compared to wild-type plants. This indicates that higher levels of CYP90D1 promote greater vegetative growth. Additionally, when these CYP90D1-overexpressing Arabidopsis plants were treated with brassinazole, a brassinosteroid biosynthesis inhibitor, their vegetative growth was restored to normal levels. This recovery suggests that the vegetative growth-promoting effect of CYP90D1 is mediated through brassinosteroid biosynthesis. These findings are significant for viticulture because they suggest that manipulating CYP90D1 expression or brassinosteroid levels could be a strategy to control vine vigor and potentially enhance berry quality. By understanding the genetic and biochemical pathways that regulate vegetative growth, viticulturists can develop more targeted approaches to manage vine development and optimize fruit production, ultimately improving the quality of wine. This research opens up new avenues for exploring how hormonal regulation can be used to fine-tune grapevine growth and berry development [53].

## 8. Future Directions

In conclusion, cytochrome P450 enzymes stand as indispensable players in improving fruit quality through plant breeding efforts. By orchestrating biochemical pathways crucial for fruit development, ripening, and quality attributes, P450 enzymes exert profound effects on sensory characteristics, nutritional content, and market appeal of fruits. Their involvement spans from the synthesis of aroma compounds and pigments to the metabolism of phytochemicals, shaping the overall fruit quality. However, the significance of P450 enzymes offers promising avenues for developing crop varieties resilient to diverse biotic and abiotic stresses, thereby bolstering agricultural sustainability and global food security. By unraveling and manipulating the metabolic networks governed byP450 enzymes, breeders can modify fruit characteristics to align with consumer preferences, adapt to shifting environmental conditions, and tackle pressing challenges in food production. To fully harness the potential of P450 enzymes in plant breeding, interdisciplinary research efforts are paramount. Integrating advancements in genomics, metabolomics, and biotechnology will empower precise manipulation of P450 enzymes mediated pathways, optimizing fruit quality traits while ensuring crop resilience and productivity. Moreover, investigating the regulatory processes governing P450 enzyme gene expression and function represents another crucial frontier in research. By elucidating the transcriptional regulation mechanisms involving transcription factors, cis-regulatory elements, microRNAs, and signaling pathways, we can gain comprehensive insights into P450 enzymes’ functionality. The integrated omics approach holds tremendous potential in crop breeding by bridging the gap between stress mitigation and achieving food security. This approach enables the construction of comprehensive models simulating plant responses to various stresses, facilitating informed breeding strategies to enhance crop productivity and resilience. Despite recent strides in the characterization and genetic engineering of plant P450 enzymes, commercialization remains a challenge. Continued research efforts aimed at unraveling the functional roles of P450 enzymes in diverse biological processes will further our understanding and drive innovation in crop breeding, ultimately benefiting agricultural sustainability and global food security.
